# Health-Related Functional Fitness within the Elderly Communities of Five European Countries: The in Common Sports Study

**DOI:** 10.3390/ijerph182312810

**Published:** 2021-12-04

**Authors:** Irimia Mollinedo-Cardalda, Manuela Ferreira, Pedro Bezerra, José María Cancela-Carral

**Affiliations:** 1 Faculty of Physiotherapy, University of Vigo, 36005 Pontevedra, Spain; 2 HealthyFit Research Group, Institute Health Research Galicia Sur (IISGS), Hospital University Complex of Pontevedra (CHOP), Sergas, University of Vigo, 36005 Pontevedra, Spain; chemacc@uvigo.es; 3 Camara Municipal of Vilanova da Cerveira, 4920-284 Vilanova da Cerveira, Portugal; manuelaferreira11@hotmail.com; 4 Escola Superior de Desporto e Lezer, Instituto Politécnico de Viana do Castelo, 4960-320 Melgaço, Portugal; pbezerra@esdl.ipvc.pt; 5 The Research Centre in Sports Sciences, Health Sciences and Human Development, 5001-801 Vila Real, Portugal; 6 Faculty of Education and Sports Science, University of Vigo, 36005 Pontevedra, Spain

**Keywords:** aging, senior fitness test, health, database, functional fitness

## Abstract

(1) Background: The purpose of this study was to analyse the functional fitness and the anthropometric values of older adults participating in the “IN COMMON SPORTS” project. (2) Methods: A total of 418 participants (eastern European Group (GEE, *n* = 124) and southern European Groups (GES, *n* = 294) have been evaluated for anthropometric characteristics and fitness. (3) Results: The GES participants presented significant differences in anthropometric values and fitness, with the best values for upper and lower limb strength and aerobic resistance, while those from the GEE presented significantly better values for lower limb flexibility. (4) Conclusion: Older adults present differences in fitness in accordance with their country of residence, with the GES having the best functional fitness.

## 1. Introduction

The European Union is one of the unique supranational entities among international organisations where citizens from 28 countries with great cultural, ethnic, artistic, and religious diversity reside. Marked differences in economic and social policies in these 28 countries come to light upon analysing economic growth using gross domestic product (GDP). Countries from southern Europe have a higher annual GDP (2018) and per capita GDP than those from eastern Europe. Per capita public expenditure on education and the promotion of healthy lifestyles is higher in countries from southern Europe than in eastern European countries [1,2]. The promotion of a healthy lifestyle is related to the practice of physical activity, but the fact is that the citizens of eastern Europe are less physically active compared to those of the South. This may also be due to climatic and environmental conditions, infrastructure and sports policies that differ greatly from one country to another [3].

Population ageing is a well-known challenge to society and science, where an increase in life expectancy is unfortunately not linked with high quality of life but, quite to the contrary, is associated with a progressive increase in disability rates in older persons [4]. Ageing in the European population presents a trend that began several decades ago. At present, 19.4% of the population is 65+ years old—an increase of 2.4 points compared to 10 years ago [5]. Therefore, a disability-free increase in life expectancy is relevant to both society and individuals. On a social level, this means a reduction in social and health care costs, whilst providing greater welfare and quality of life to citizens [4]. On an individual level, it implies better functionality and independence of these senior citizens.

Ageing is associated with loss of neuromuscular function, which leads to a reduction in strength [6,7] and loss of muscular mass [8,9], decreased walking speed, greater risk of falls, and reduced capacity to perform the activities of daily life. All these contribute to loss of independence and are detrimental to the quality of life of older adults [10].

The World Health Organization (WHO) uses the term “Active Ageing” to denote the process that achieves this goal [11]. The approach to ageing recognizes the human rights of senior citizens, as well as the United Nations principles of independence, participation, dignity, support, and realization of one’s desires. The WHO [12] recommends that adults over 65 years of age take part in at least 150 min of moderate-intensity aerobic physical activity throughout the week, maintain functionality and quality of life of the ageing population, and improve cardiorespiratory and muscular fitness, bone, and functional health, and reduce the risk of non-communicable diseases, depression and cognitive decline. The European Union [13] data shows that in most countries, adults over 75 years of age take part in less physical exercise/week compared to the 65–74 age range, resulting in increasing levels of illness and weakness among older adults.

For this reason, projects that promote and develop physical activity for senior citizens such as “Living with vitality” [4], “Vivifrail” [14] and the comprehensive plan to promote sport and physical activity in older adults [15] have been created over the last two decades. The European Erasmus + “IN COMMON SPORTS 2018–2020” was born within this framework with the participation of countries such as Hungary, Bulgaria, Italy, Portugal and Spain. Its aim is to promote the active ageing of older adults through the training and practice of competitive adapted sports (basketball, volleyball, soccer, athletics, and water polo) and to ascertain whether competition increases the greater adhesion of older adults to the programs. Before starting an intervention program with older adults, it is necessary to know their functional fitness (strength, endurance, flexibility, and balance) to individualize the training program and be able to compare its effect [15]. We also assessment anthropometrics parameters (body mass index and waist hip ratio) and cognitive parameters (cognitive impairment and level of motivation). In this study, this assessment is very important due to the different socio-economic status of the countries involved: Bulgaria and Hungary vs. Portugal, Italy, and Spain [3].

Therefore, the research questions of this study have been: 1. Are there differences in the functional fitness and the anthropometric values of older adults depending on the country of residence (eastern vs. southern Europe)? 2. Gender can be a factor that conditions these differences? 3. Can the amount of physical exercise developed per week (<150 min/week vs. ≥150 min/week) make a difference in the functional fitness and the anthropometric values of older adults?

## 2. Materials and Methods

### 2.1. Participants

A sample of 418 participants: 318 women (76%; 69.29 ± 7.62) and 100 men (24%; 68.97 ± 7.09) were recruited for the European Erasmus+ “IN COMMON SPORTS” project in which five European countries participated: Bulgaria, Hungary, Portugal, Italy and Spain. This project promotes physical activity in over 60 years of age residents of Europe, in accordance with the ethical principles of the Declaration of Helsinki. Table 1 shows the descriptive characteristics of the sample divided into two groups: eastern Europe (Bulgaria and Hungary) and southern Europe (Italy, Spain, and Portugal). The inclusion criteria taken into account were the same as those used when implementing the European project: (1) over 60 years of age, (2) residents in the city where the program is implemented or in its vicinity, (3) have a medical certificate to participate in the “IN COMMON SPORT” training program, and (4) ability to walk continuously of a minimum of 6 min, (5) lacking in moderate cognitive impairment, (6) do not play competitive sports. The ethical standards contained in the Declaration of Helsinki were followed in this study, and this study was approved by the ethics committee of the Polytechnic Institute of Viana do Castelo (IPVC-ESDL180417). In addition, all participants signed an informed consent prior to participation in the program. The “IN COMMON SPORTS” program recruited participants voluntary through the websites and press advertisements that each municipality spread, and where the project and the steps for enrolment were announced.

### 2.2. Demographic Measures

The demographic data collected included age, gender, and place of residence of the participants on an ad hoc record sheet. Information recorded included quantity; type of activity/exercise carried out in minutes/week by participants during a typical week.

### 2.3. Anthropometric Measures

The height (cm) of the patients was collected using a 1.0 mm Handac model stadiometer, while the weight (kg), body mass index (kg/m^2^), and body fat (%), were recorded using the Tanita model MC-780MA body composition analyser [16]. Hip and waist circumferences were measured using a 6 mm Lufkin W606PM anthropometric tape, and the waist-to-hip ratio (WHR) was found using the “Waist/Hip” formula. The WHR is used as an indicator of health and the risk of developing serious health disorders, as well as evaluating abdominal obesity [17].

### 2.4. Handgrip

The Handgrip is an instrument that evaluates the strength of upper limbs through manual pressure. The test is performed with the participant standing with the elbow in 90° flexion and slightly separated from the trunk [18]. Three measurements are taken for each limb and the average is calculated. In this study we used the hydraulic handgrip model SH5001 from Saehan Corporation, which measures the force in kilograms.

### 2.5. Senior Fitness Test

The Senior Fitness tests is a battery of tests designed to assess the functional fitness of older adults. This test assesses the physiological capacity for carrying out normal daily activities independently and safely without the appearance of fatigue. Test validity has been published by Rikli and Jones [19]. The test consists of six measures of functional fitness, although we only use five as the arm curl test is replaced by the Handgrip test, and both these tests evaluate the strength of the upper limbs.

Chair sit and reach: This test measures lower body flexibility. The participant sits on the edge of a chair (placed against a wall for safety). One foot must remain flat on the floor. The other leg is extended forward with the knee straight, heel on the floor, and ankle bent at 90°. The participants placed one hand on top of the other with tips of the middle fingers even. The participants were told to inhale and, when exhaling, to try to touch their toes by bending at the hips. Participants had to keep their backs straight and their heads upright. Participants had to avoid bouncing or rapid movements and never stretch to the point of pain. The knee had to be kept straight during the stretch for 2 s to measure the distance between the tips of the toes to the feet. If participants touched the feet with their fingertips, the score was zero. If they did not touch, the distance between the fingers and toes was averaged (a negative score), and if they could reach further than their toes, this distance was measured (a positive score). Participants performed the movement twice.

Back scratch: This test measures the overall range of shoulder movement. Participants performed the test in the standing position. They placed one hand behind their head and back by reaching back over their shoulder and reach as far as possible down the middle of the back with the palm touching the body and the fingers directed downwards. They placed the other arm behind their back with the palm facing outward and fingers upward and reached up as far as possible while attempting to touch or overlap the middle fingers of both hands. An assistant ensured that the participants’ fingers were aligned and measured the distance between the tips of the middle fingers. If the fingertips touched, the score was zero. If they did not touch, the distance between the fingertips was measured (a negative score), and if they overlapped, this overlapping distance was measured (a positive score). The participants had two attempts to understand how the test worked, then carried out two more attempts, which were measured. The test was halted if the participants experienced any pain.

The 30 s chair stand test: This test assesses lower body strength and stamina. The participants crossed their hands over their chests, and sat down and stood up from a chair as many ties as possible over a period of 30 s. The number of repetitions achieved during this time was recorded.

The eight foot up and go: This test assesses speed, agility, and dynamic balance. Starting from sitting, the participant will have to get up and walk to the cone, turn around and sit down again. The cone will be 2.44 m from the chair. The participant will perform the test twice and the score will be the average of the two times.

The six min walk: This test measures aerobic fitness. The walking course is laid out in a 50 yard (45.72 m) rectangular area (dimensions 45 × 5 yards), with cones placed at regular intervals to indicate the distance walked. The aim of this test is to walk as quickly as possible for six minutes to cover as much ground as possible. Participants are set their own pace (a preliminary trail is useful to practice pacing) and are able to stop for a rest if they desire.

The protocols of the tests were supervised by a group of graduates in physical therapy and physical activity sciences, experts in older adults. Each country had its own group of experts, but with a common consensus to avoid bias. The reliability between the different groups of testers was calculated using the Cohen’s Kappa coefficient. This value was between 0.68–0.79 for the questionnaires and between 0.88–0.96 for the physical tests.

### 2.6. Procedure

The representatives of each country belonging to the European project “IN COMMON SPORTS”, contacted the participants to carry out a first assessment of the individuals in March 2018. Within one week, all participants were summoned simultaneously in all countries (Italy, Spain, Portugal, Bulgaria, Hungary). The assessments were made in sports centres (gym, sports hall) where the subjects carried out their training, and the data were dumped on the common SPSS statistical system for all countries, where the different statistical analyses were performed. In the statistical analysis, the sample was divided into two groups, participants belonging to eastern European countries (Bulgaria and Hungary) and southern European countries (Spain, Portugal and Italy).

### 2.7. Statistical Analysis

Descriptive statistics were performed for all measures of the participants. Between groups, differences were evaluated using student t for independent data, segmenting such analysis by gender and physical activity level (≥150 min/week, <150 min/week) and place of residence. Previously, the sample was checked for normality, through the Kolmogorov–Smirnov test. The relationship between BMI and other measured variables was tested for linearity with Pearson’s product-moment correlation coefficients. Stepwise multivariate regression analyses were used to model log-transformed BMI, and the model was adjusted according to gender and country. The significance level was set at 0.05. All data analyses were performed with the Statistical Package for the Social Sciences Version 24 (IBM-SPSS, Chicago, IL, USA).

## 3. Results

A total of 418 older adults participated in the first assessment of the project, being divided into two groups according to European location, culture, and lifestyle, these being: eastern European countries (Hungary and Bulgaria), and southern European countries (Spain, Portugal and Italy). As can be seen in Table 1, the eastern Europe group (GEE) is made up of 124 people (85% female) aged 68.63 ± 7.33 years who take part in 133.90 ± 8.12 min/week of physical activity, while the Southern Europe group (GES) is made up of 294 people aged (73% female) 69.63 ± 9.04 years who undertake 136.63 ± 8.34 min/week of physical activity. These groups are homogeneous in terms of age and physical activity. Regarding anthropometric measurements, it was observed that the participants of the GES are significantly lower, and less heavy than the GEE, showing a body mass index and a percentage of body fat significantly lower than the GEE. Likewise, in the measurement of circumferences it is shown that the GES presents significantly lower values for the waist and hip circumferences than the GEE, but if we observe the WHR index, the GEE presents a significantly lower value.

Table 2 shows the division of the sample by group (GEE vs. GES) and by gender (male vs. female), where it can be seen that in the male older adult population the amount of physical activity they take part in per week is significantly lower in the GEE than in the GES, while in women, no significant differences are observed, although the trend of the male older adult population continues. As for the physical data, in the male population significant differences are observed in the strength of upper limbs and in dynamic balance, with men belonging to GES demonstrating greater strength and better balance. As for women, the data follow the same lines as in the male population, GES presents significantly higher values of strength and balance than GEE.

Table 3 shows a division by group (GEE vs. GES) by gender (female vs. male) and by the amount of weekly physical activity performed (≥150 min/week vs. <150 min/week). This table shows a comparison between intra- and inter-groups, the latter depending on the physical activity performed. Therefore, in the intra-group analysis, referring to the EEG, who perform <150 min/week, women had significant differences in strength and flexibility of lower limbs, aerobic resistance, and dynamic balance than those who performed physical activity. While men who practice <150 min/week of physical activity showed lower strength of the right hand and lower limb, less aerobic resistance, balance, and flexibility of the lower limbs than those who carry out ≥150 min/week of physical activity. Referring to the GES, people who carry out <150 min/week, women showed significant differences in strength in the left hand and lower limbs, aerobic resistance, and balance than those who carried out ≥150 min/week of physical activity. While men who practice <150 min/week of physical activity showed lower strength of the left hand and lower limbs strength, aerobic and flexibility resistance of upper and lower limbs than those who carried out ≥150 min/week of physical activity.

In the inter-group comparison relating to the amount of weekly physical activity performed, it is observed that the GES female presents significant differences in upper and lower limb strength, and balance, with better values than the GEE in the population that carries out ≥150 min/week. Likewise, in the population that carries out ≥150 min/week the GES female shows that people who practice <150 min/week present significantly different results, with higher values for strength-both of upper and lower limbs, and also of balance. It should be noted that there are significant differences in terms of age, with a significantly younger population undertaking less physical activity in GEE than in GES. In the case of men, significant differences were observed in men who practice <150 min/week, showing greater strength in upper and lower limbs, and in balance for GES. While those who carried out ≥150 min/week showed significant differences with greater strength upper limbs and balance for the GES.

Table 4 shows a correlation between BMI and the fitness condition variables, all of which presented significant results. The variables with the highest correlation are the back scratch, 30 s chair stand, 8 foot up and go, and chair sit and reach.

The stepwise regression analysis (adjusted according to gender and country) showed that four variables (Left handgrip, 30 s chair stand, 8 Foot up and go and Back scratch) were significantly associated with the BMI. Together, these variables explained 22% (R = 0.42, R^2^ _adj_ = 0.218, F (4.414) = 18.607, *p*, 0.001) of the variance in the BMI. Together, these variables formed the following BMI prediction model (y = 25.931 + 0.049 x_1_ − 0.133 x_2_ + 0.334 x_3_ − 0.109 x_4_) where “y” is BMI (kg/m^2^), “x_1_” is Left handgrip (kg), “x_2_” is 30 s chair stand (*n*), x_3_ is 8 Foot up and go (s), and “x_4_” is Back scratch (cm).

## 4. Discussion

Knowing the effects that physical inactivity causes on the functional fitness is key to defining correct policies for each of the country, thus improving the health status and quality of life of older adults and reducing health care cost.

The socio-cultural and economic contexts in which the European population carries out its daily activities are key and show the way that the society of the future will develop. At present, the European population over 60 years of age shows that country differences are reflected in their social and economic policies, the welfare state, food, education, climate, etc [13]. As mentioned in the introduction, eastern European countries have a lower annual and per capita GDP than southern European countries, and this fact has repercussions on education, health and, in short, on the welfare of citizens. Due to this, it is necessary and of vital importance to develop programs such as “IN COMMON SPORTS”, to motivate and provide resources for older adults to continue to remain physically active throughout their lives, as European data [13] show that from the age of 75, people stop being physically active. This is why programs such as these are necessary to keep older adults active, functional and autonomous, both to ensure their quality of life and to save costs for the State [20].

As for anthropometric variables, inhabitants of eastern Europe are higher than the inhabitants of southern Europe, and this is in line with other previous studies [21]. Older adults living in southern Europe have a lower fat content and WHR ratio than those living in the east, which may be due to diet, since Southern Europe advocates a Mediterranean diet with abundant vegetables, fruits, and fish [22]. Likewise, the climate can also be an important variable in this case, since the climate in southern Europe favours outdoor activities.

Differences were also observed in terms of fitness capacities according to the region and the hours of physical exercise per week. Firstly, it should be noted that one of the most significant phenomena of aging is sarcopenia (loss of muscle mass and strength), thus bringing about the loss of functional abilities in such vital gestures as climbing stairs or getting up from a chair [23]. The decline in both muscle mass and strength that occurs with aging is well documented. Thus, muscle function is of greater importance than muscle mass, validating manual dynamometry as an indicator of functionality in older adults [24]. In fact, the study by Mancilla et al. [18] establishes the degree of independence of the participant as a function of the levels of strength collected by means of a dynamometer, according to the age range, although it should be highlighted that the sample of this study is self-validating regardless of the region and, if we take this study as a reference. As for the strength of the lower limbs, Jones and Rikli [25], creators of the Senior Fitness Test battery, established normative values, which the average of the sample complies with. Although both groups, GES and GEE, comply with the normative values of strength, it is necessary to emphasize that people who live in southern Europe present a greater muscular strength of both upper and lower limbs, which may allow greater functionality, and thus, favour the quality of life of the participant.

It should be noted that authors such as Castañeda, Gómez, Avellaneda, Caballero, and Delgado [26] relate lower limb strength with balance and therefore a lack of it with the risk of suffering a fall. Cadore et al. [27] suggests that muscle strength works accompanied by high intensity activities, aimed at improving muscle power, improves walking speed, the ability to get up from a chair, balance, and reduces the incidence of falls. García-Flores, Rivera-Cisneros, Sánchez-González, Guardado-Mendoza, and Torres-Gutiérrez [28] state that the most important components associated with balance are muscle strength and walking speed. These are the factors responsible for maintaining the autonomy of older adults during aging, which is also reflected in this study, where the population of southern Europe shows better levels of strength and also balance, in relation to that of eastern Europe, regardless of gender.

It has also been observed that there is a relationship between BMI and physical fitness variables: the higher the BMI, the lower the strength, balance, flexibility, and aerobic endurance. However, this can only be explained in 22% of the sample. Ho et al. [29] analysed the associations between health-related physical fitness performance and overweight/obesity risk among Taiwanese healthy older adults. The results of this study have revealed the existence of an association between BMI levels and physical fitness, indicating that the older people who present a poor level of physical condition have a greater risk of having a high BMI (overweight obesity). The findings obtained by Ho et al. [29] also indicate that the BMI is unable to predict the strength levels of the lower extremities, muscular endurance, and aerobic physical fitness, as older adults present different body densities and BMI values cannot wholly reflect the proportions of an individual’s muscles and fat [29]. These results justify the low percentage of representativeness obtained in our study (22%) when performing the stepwise regression analysis (adjusted according to gender and country) to determine the BMI based on physical fitness.

Finally, it should be noted that in this study, 50% of the population over 60 years of age carried out physical exercise for more than the 150 min per week, as recommended by the WHO (2020), regardless of the region of residence. Eurostat (2019) stratified its study by age groups (50–64; 65–74; +75) showing the percentage of the population that carries out more than 3 h per week of physical exercise. Spain presented the highest values (48%, 51%, and 32%, respectively), followed by Italy (25%, 32%, and 20%), Bulgaria (32%, 30%, and 16%), Portugal (23%, 25%, and 16%), and Hungary (25%, 22%, and 15%). As can be seen in this study, from the age of 75 onwards, the percentage of people who do physical exercise for at least 3 h a week is significantly reduced. That is why it is necessary to implement projects, such as “IN COMMON SPORTS”, to offer the opportunity and motivation to the older adult population to do physical exercise, which will benefit the individual, as they will maintain their autonomy, as well as benefitting the State by saving health and dependency costs.

In addition, this study supports the WHO [12] which establishes the duration of weekly physical exercise that older adults must do in order to maintain their functionality, autonomy and thus an active and quality lifestyle. There are significant differences in limb strength and balance when the amount of weekly exercise is considered, although there are also significant differences in upper limb strength and aerobic capacity in Southern Europe. This may be because the type or load of physical exercise that this population does is not the same. Therefore, in future research it would be advisable to establish what type and load of physical exercise is advisable in order to establish changes and improvements in the functional fitness of that population.

This research presents a series of limitations that we will now indicate. The first limitation to highlight is the cross-sectional design, which makes it impossible to track the functional fitness of older adults. Another limitation is that the sample used was chosen for convenience and is not representative of the population of each country, although it may indicate a trend. The third limitation is the small size of the sample used. The number of men who have participated in the study is very small. The researchers have decided to keep the analysis planned, as these data reflect the reality of sports physical practice by men. The fourth limitation is related to the evaluation team, which despite using the same protocols, was different for each country, and an inter observer bias may appear.

Although this study has these limitations, this study suggested differences in anthropometric and functional fitness parameters between the population over 60 years of age living in Eastern and Southern European countries. Future lines of research should be directed towards testing how the different physical condition variables behave according to the type of physical activity undertaken.

The findings of this research have generated a series of recommendations to be carried out in physical training for older adults: 1. The sociocultural and economic context of the participants determines their physical fitness and body composition. The results recommend that in eastern European countries physical exercise programs focus on developing strength, flexibility, endurance and lowering the percentage of body fat. 2. Gender of the participants interferes with the physical fitness. We recommend that women living in eastern European countries participate in physical exercise programs to improve upper and lower limb strength levels, agility, and dynamic balance. Men should participate in physical exercise programs that improve upper body strength, agility, and dynamic balance levels. 3. The amount of weekly physical exercise is a factor that influences the physical fitness of older adults. Physical exercise programs must have a minimum duration of 150 min/week.

## 5. Conclusions

It is necessary to implement physical exercise programs in people over 60, especially in eastern European countries, as well as the amount of weekly physical exercise carried out according to WHO criteria, in order to keep older adults active and autonomous.

## Figures and Tables

**Table 1:** Comparative analysis of demographic, anthropometric and fitness measures of residence in eastern Europe and southern Europe.

	Eastern Europe *n* = 124		Southern Europe *n* = 294	
	Mean	SD	Mean	SD
Demografic data				
Age (years)	68.63	7.33	69.63	9.04
Gender (female, %)	82.25	-	73.46	-
Physical activity (min/week)	133.90	8.12	136.63	8.34
Antropometric data				
Height (cm)	161.43	8.16	150.33	39.91 **
Weight (kg)	76.59	14.09	72.35	14.01 **
Body mass index (kg/m^2^)	29.42	5.20	27.98	5.35 *
Fat body (%)	36.98	6.80	31.25	8.61 **
WHR	0.87	0.07	0.90	0.13 *
Fitness data				
Right handgrip (kg)	23.83	8.43	29.15	9.13 **
Left handgrip (kg)	20.46	9.21	27.79	10.31 **
30 s chair stand (*n*)	14.58	3.25	17.98	5.88 **
6 min walk (m)	498.96	217.90	533.16	121.02 *
Chair sit and reach (cm)	2.41	7.91	0.34	10.26 *
8 Foot up and go (s)	8.02	2.64	8.96	38.53
Back scratch (cm)	−9.26	9.61	−9.15	13.89

* WHR = Waist to hip ratio; ** *p* < 0.001; * *p* < 0.05.

**Table 2:** Results for physical fitness tests for gender.

	Male *n* = 100				Female *n* = 318			
	Eastern Europe *n* = 22		Southern Europe *n* = 78		Eastern Europe *n* = 102		Southern Europe *n* = 216	
	Mean	SD	Mean	SD	Mean	SD	Mean	SD
Fitness data								
Right handgrip (kg)	29.46	9.98	39.09	9.01 **	21.56	6.74	25.61	6.65 **
Left handgrip (kg)	26.02	11.97	37.76	9.36 **	17.86	6.95	24.26	9.23 **
30 s chair stand (*n*)	15.89	3.71	17.11	4.84	14.25	3.27	16.11	5.38 **
6 min walk (m)	519.36	222.28	528.26	93.35	469.85	226.36	481.73	97.55
Chair sit and reach (cm)	−3.21	6.38	−2.47	10.78	2.23	7.72	0.77	9.28
8 Foot up and go (s)	8.34	2.99	6.73	2.13 *	8.59	2.54	7.23	2.20 **
Back scratch (cm)	−13.61	13.54	−13.87	15.38	−9.56	8.74	−9.40	13.18

** *p* < 0.001; * *p* < 0.05.

**Table 3:** Physical fitness results by gender in relation to the physical activity level.

	Female							
	Eastern Europe (*n* = 102)				Southern Europe (*n* = 216)			
	≥150 min/Week *n* = 48		<150 min/Week *n* = 54		≥150 min/Week *n* = 107		<150 min/Week *n* = 109	
	Mean	SD	Mean	SD	Mean	SD	Mean	SD
**Fitness data**								
Right handgrip (kg)	22.70	4.83	20.42	4.28	26.53	11.78 ^##^	24.69	8.72 ^##^
Left handgrip (kg)	17.98	6.03	17.74	5.05	26.15	12.06 ^##^	22.37	14.6 *
30 s chair stand (*n*)	16.64	2.54	11.86	2.72 *	17.55	5.42	14.67	5.69 *^##^
6 min walk (m)	583.54	137.96	356.16	100.15 *	595.3	80.42	368.16	114.02 *
Chair sit and reach (cm)	4.39	11.69	0.07	6.38 **	0.96	7.43 ^#^	0.58	9.93
8 Foot up and go (s)	7.60	2.44	9.58	2.57 *	6.37	1.39 ^##^	8.09	2.8 *
Back scratch (cm)	−9.45	6.27	−9.67	5.41	−8.1	10.88	−10.7	13.07
	**Male**							
	Eastern Europe (*n* = 22)				Southern Europe (*n* = 78)			
	≥150 min/week *n* = 12		<150 min/week *n* = 10		≥150 min/week *n* = 47		<150 min/week *n* = 31	
	Mean	SD	Mean	SD	Mean	SD	Mean	SD
**Fitness data**								
Right handgrip (kg)	30.78	8.03	28.14	3.18 *	39.97	7.66 ^##^	38.21	9.34 ^##^
Left handgrip (kg)	26.48	11.59	25.56	5.31	41.15	7,32 ^##^	34.37	9.90 *^##^
30 s chair stand (*n*)	17.4	3.50	14.38	2.88 *	19.01	4.50	15.21	5.01 *^##^
6 min walk (m)	636.15	126.78	402.57	111.87 *	644.82	98.50	411.7	86.26 *
Chair sit and reach (cm)	−1.75	6.73	−4.67	7.46 *	−1.56	10.95	−3.38	10.69 *
8 Foot up and go (s)	7.42	3.38	9.26	3.37 *	6.25	1.37 ^#^	7.21	2.54 ^##^
Back scratch (cm)	−13.85	15.13	−13.37	13.21	−12.92	16.86	−14.82	14.11 *

Obs: ** *p* < 0.001, * *p* < 0.05, intra-group differences (Eastern Europe; Southern Europe); ^##^ *p* < 0.001, ^#^ *p* < 0.05, inter-group differences (eastern vs. southern Europe).

**Table 4:** Correlation between the Fitness variables and BMI.

Variable	rs	*p*	*n*
Right handgrip (kg)	−0.106	0.018	418
Left handgrip (kg)	−0.083	0.046	418
30 s chair stand (*n*)	−0.346	0.001	417
6 min walk (m)	0.151	0.002	416
Chair sit and reach (cm)	−0.255	0.001	417
8 Foot up and go (s)	0.316	0.001	416
Back scratch (cm)	−0.425	0.001	418

## Data Availability

http://www.olympics4all.eu/index.php, accessed date 3 December 2021.
