# Correction: Mollinedo-Cardalda et al. Health-Related Functional Fitness within the Elderly Communities of Five European Countries: The in Common Sports Study. *Int. J. Environ. Res. Public Health* 2021, *18*, 12810

**DOI:** 10.3390/ijerph20126186

**Published:** 2023-06-20

**Authors:** Irimia Mollinedo-Cardalda, Manuela Ferreira, Pedro Bezerra, José María Cancela-Carral

**Affiliations:** 1 Faculty of Physiotherapy, University of Vigo, 36005 Pontevedra, Spain; 2 HealthyFit Research Group, Institute Health Research Galicia Sur (IISGS), Hospital University Complex of Pontevedra (CHOP), Sergas, University of Vigo, 36005 Pontevedra, Spain; 3 Camara Municipal of Vilanova da Cerveira, 4920-284 Vilanova da Cerveira, Portugal; 4 Escola Superior de Desporto e Lezer, Instituto Politécnico de Viana do Castelo, 4960-320 Melgaço, Portugal; 5 The Research Centre in Sports Sciences, Health Sciences and Human Development, 5001-801 Vila Real, Portugal; 6 Faculty of Education and Sports Science, University of Vigo, 36005 Pontevedra, Spain

## Text Correction

There was an error in the original publication [1]. The ethical code is missing from section Materials and Methods.

A correction has been made to Section 2.1. Participants, lines 12–14:

The ethical standards contained in the Declaration of Helsinki were followed in this study, and this study was approved by the ethics committee of the Polytechnic Institute of Viana do Castelo (IPVC-ESDL180417).

The authors state that the scientific conclusions are unaffected. This correction was approved by the Academic Editor. The original publication has also been updated.

## References

[1] Mollinedo-Cardalda I., Ferreira M., Bezerra P., Cancela-Carral J.M. (2021). Health-Related Functional Fitness within the Elderly Communities of Five European Countries: The in Common Sports Study. Int. J. Environ. Res. Public Health.

